# *EGFR*敏感突变阴性非小细胞肺癌脑转移患者临床特征

**DOI:** 10.3779/j.issn.1009-3419.2020.102.48

**Published:** 2021-01-20

**Authors:** 以香 朱, 烨 张

**Affiliations:** 100021 北京，国家癌症中心/国家肿瘤临床医学研究中心/中 国医学科学院北京协和医学院肿瘤医院放疗科 Department of Radiation Oncology, National Cancer Center/National Clinical Research Center for Cancer/Cancer Hospital, Chinese Academy of Medical Sciences and Peking Union Medical College, Beijing 100021, China

**Keywords:** 肺肿瘤, 脑转移, 突变阴性, 临床特征, Lung neoplasms, Brain metastases, Mutation negative, Clinical features

## Abstract

除少见的间变性淋巴瘤激酶（anaplastic lymphoma kinase, *ALK*）及原癌基因-1受体酪氨酸激酶（*c*-*ros* oncogene 1-receptor tyrosine kinase, *ROS1*）阳性敏感融合外，非表皮生长因子受体（epidermal growth factor receptor, *EGFR*）敏感突变的非小细胞肺癌（non-small cell lung cancer, NSCLC）脑转移患者目前无有效的全身治疗药物，整体预后较差。由于传统药物血脑屏障透过率低，脑转移的局部治疗尤其是放疗具有非常重要作用。为了更好地认识*EGFR*突变阴性NSCLC脑实质转移的特点，本文从脑转移的发病率、发病时间、发病部位、病灶数目及大小、发病症状、治疗疗效和病情演变等方面综述了*EGFR*突变阴性NSCLC脑实质转移的临床特征以及治疗，为脑实质转移局部治疗的介入时机以及局部治疗技术选择提供参考。

## Abstract (en)

In addition to rare incidence of anaplastic lymphoma kinase (*ALK*) and *c-ros* oncogene 1 receptor kinase (*ROS1*) positive patients, patients with brain metastases of non-small cell lung cancer (NSCLC) without epidermal growth factor receptor (*EGFR*) sensitive mutation have no effective systemic therapy at present, and the overall prognosis is poor. Since the low blood-brain barrier permeability of chemotherapy drugs, the local treatment plays an important role in brain metastases. To understand the clinical characteristics and treatment of brain parenchymal metastases in patients with NSCLC and *EGFR* mutation negative, we reviewed the incidence, onset time, site, numbers, size, symptom, therapeutic effect and disease evolution in them, which can provide reference for interventional timing and local treatment technology selection of local treatment for brain parenchymal metastases.

非小细胞肺癌（non-small cell lung cancer, NSCLC）是肺癌最常见的病理类型，7.4%-10%的NSCLC患者初诊合并脑转移，30%-50%的患者病程中会发生脑转移^[1-5]^。敏感基因的发现以及相应的靶向药物出现是近些年肺癌的重大进展，NSCLC中最常见的敏感基因突变类型为表皮生长因子受体（epidermal growth factor receptor, *EGFR*）突变，占10%-40%^[6, 7]^，其他相对常见的间变性淋巴瘤激酶（anaplastic lymphoma kinase, *ALK*）及原癌基因-1受体酪氨酸激酶（*c-ros* oncogene 1-receptor tyrosine kinase, *ROS1*）扩增/融合发生率较低，仅为3%-5%^[8, 9]^。有研究报道了*EGFR*敏感突变的NSCLC合并脑转移的发病特征，但是缺乏对阴性患者的深入探索。目前，除外*ALK*等敏感基因外，大部分非*EGFR*敏感突变的肺癌脑转移患者无有效的治疗药物，整体预后较差。放疗在脑转移的治疗具有重要作用。为了更好地认识*EGFR*敏感突变阴性NSCLC脑实质转移的特点，本文综述了*EGFR*敏感突变阴性的NSCLC脑实质转移的临床特征以及全身药物治疗疗效，并与*EGFR*敏感突变的NSCLC脑实质转移的特征进行对比，为脑实质转移局部治疗的介入时机以及局部治疗技术选择提供参考。

## 1 发病率

Hsu等^[10]^对2010年-2012年初治的422例Ⅳ期*EGFR*突变阴性非鳞NSCLC患者分析发现，初诊脑转移的发生率为25.1%（106/422），与*EGFR*突变阳性患者相近（30.6%, *P*=0.36），治疗后1年和3年累积脑转移发生率分别为28.1%和28.2%，低于阳性患者的39.0%（*P*=0.041）和39.2%（*P*=0.038）。Wang等^[11]^的研究结果与上述相似，在2005年-2013年的1, 207例*EGFR*突变阴性晚期非鳞NSCLC患者中，初诊脑转移发生率为24.6%（297/1, 207），1年和3年累积脑转移率分别为29.7%和35.8%，亦低于阳性患者的35.3%和46.2%（*P*=0.008）。Nishiyama等^[12]^也回顾性分析了2007年-2011年既往治疗过的203例晚期*EGFR*突变阴性NSCLC患者，指出累积脑转移的发生率为28.6%（58/203）。略有不同的是，Baek等^[13]^分析2010年-2013年确诊的259例晚期NSCLC，发现*EGFR*突变阴性患者的同时性脑转移（14.5% *vs* 27.4%, *P* < 0.009）和累积脑转移（21.5% *vs* 37.0%, *P* < 0.006）的发生率均低于*EGFR*突变阳性患者。此外，Chen等^[14]^分析了2010年-2016年291例晚期NSCLC，发现*EGFR*和*ALK*均阴性的患者脑转移的发生率为16.5%（32/194），显著低于*ALK*阳性患者（26.5%, *P*=0.038）。整体而言，*EGFR*突变阴性NSCLC初诊脑转移的发生率与阳性患者相似，但治疗后累积脑转移发病率较阳性患者低。

## 2 发病时间

目前国际上根据脑转移发生的时间分为同时性和异时性脑转移，从原发肿瘤确诊至3个月内出现的脑转移定义为同时性脑转移，3个月后定义为异时性脑转移。张永芹等^[15]^研究显示*EGFR*突变阴性患者从原发病灶确诊到发生脑转移的平均时间为7个月，与*EGFR*突变阳性患者类似（7个月*vs* 9.6个月，*P* < 0.05）。Baek等^[13]^研究数据与上述结果相似，在186例*EGFR*突变阴性患者中，初诊性脑转移占14.5%（27/186），异时脑转移占7.0%（13/186），其中异时脑转移的出现时间为8.8个月。略有不同的是，Eichler等^[16]^报道的异时脑转移的发生时间较上述时间长，*EGFR*突变阴性与阳性患者初诊到发生脑转移的时间分别为14个月和19个月（*P*=0.27）。

综上，*EGFR*突变阴性患者从确诊肺癌到发生脑转移的时间约为7个月-14个月，与阳性患者无明显差异。

## 3 发病部位

张永芹等^[15]^对27例*EGFR*突变阴性患者的脑转移部位进行了详细描述，病灶位于幕上、幕下和幕上幕下同时累及者分别为59.3%（16/27）、3.7%（1/27）和33.3%（9/27），还有1例患者同时侵犯颅骨。累及幕上者，以顶叶（22.2%, 6/27）和额叶（18.5%, 5/27）最为常见，颞叶和枕叶相对较少，未见脑实质伴脑膜转移者。Takano等^[17]^指出*EGFR*突变阴性患者最常见的脑转移部位是额叶，之后依次为顶叶、小脑、枕叶和颞叶。并且脑转移灶到大脑表面的平均距离为1.61 cm（范围：1.52 cm-1.71 cm），大于*EGFR*外显子21突变患者（1.24 cm，范围：1.09 cm-1.39 cm）（*P*=0.000, 1）。另外，Hsu等^[18]^的研究还发现*EGFR*突变阴性患者出现粟粒状脑转移的发生率为1.7%，显著低于*EGFR*突变阳性患者（8.5%, *P*=0.035）。总体来说，*EGFR*突变阴性NSCLC患者脑转移病灶多见于幕上，常发生于额叶和顶叶，到大脑表面的距离大于*EGFR*外显子21突变患者。

## 4 发病数目

Eichler等^[16]^对52例*EGFR*突变阴性NSCLC脑转移患者的颅内病灶数目进行了分析，发现脑转移数量为1个、2个-3个、 > 3个和不详分别为35%（18/52）、35%（18/52）、29%（15/52）和2%（1/52）。Sekine等^[19]^发现*EGFR*突变阴性患者的中位脑转移数量为3个（范围：1个-38个），与*EGFR*外显子19突变（中位数：5个，范围：1个-100个）相比，*EGFR*突变阴性患者较少出现多发脑转移（*P*=0.024）。Balasubramanian等^[20]^对254例目前可识别的驱动基因均阴性的NSCLC伴脑转移患者的脑转移数进行了汇总，中位的脑转移数为2个，其中脑转移数为1个、2个-3个和 > 3个者分别46%（118/254）、28%（70/254）和26%（66/254）。因此，与*EGFR*突变阳性患者相比，*EGFR*阴性患者脑转移灶数量相对局限，较少出现多发脑转移（脑转移数量多≤3个）。

## 5 病灶大小

Sekine等^[19]^运用磁共振成像（magnetic resonance imaging, MRI）对102个脑转移的大小和瘤周水肿进行了测量，结果显示*EGFR*突变阴性患者的中位脑转移直径为1.10 cm（范围：0.29 cm-6.20 cm），与*EGFR*外显子19突变（中位值0.71 cm，范围：0.30 cm-2.00 cm）相比脑转移灶整体偏大（*P*=0.001, 6）。此外，*EGFR*突变阴性患者的中位瘤周水肿范围为0.88 cm（范围：0.00 cm-7.66 cm），也较*EGFR*外显子19突变患者（0.03 cm，范围：0.00 cm-3.60 cm）的瘤周水肿范围更广（*P*=0.003, 6）。该研究还指出*EGFR*突变阴性患者的脑转移灶合并瘤周水肿范围 < 1.0 cm、 < 2.0 cm、 < 3.0 cm的比例分别为37.0%（20/54）、53.7%（29/54）和61.1%（33/54）。与*EGFR*外显子19突变患者相比，*EGFR*突变阴性患者脑转移合并瘤周水肿范围 < 1.0 cm（*P*=0.001）、 < 2.0 cm（*P*=0.000, 3）和 < 3.0 cm（*P* < 0.000, 1）的比例更低。不同的是，Takaco等^[17]^的研究则发现在*EGFR*突变阴性的肺腺癌患者中，脑转移的平均直径是0.56 cm（范围：0.36 cm-0.87 cm），较前者报道的小。整体来说，*EGFR*突变阴性患者脑转移多大于1.0 cm，且对比*EGFR*敏感突变患者的脑转移直径更大，瘤周水肿范围更广。

## 6 发病症状

Sekine等^[19]^对31例*EGFR*突变阴性NSCLC脑转移患者的初诊症状进行了分析，依次为：呼吸系统症状（51.6%, 16/31）、神经系统症状（25.8%, 8/31）、疼痛（9.7%, 3/31）、胸部影像学异常（9.7%, 3/31）、食欲下降（3.2%, 1/31）等，在神经系统症状中包括瘫痪5例（62.5%）、癫痫1例（12.5%）、头痛1例（12.5%）和运动障碍1例（12.5%）。此外，张永芹等^[15]^的研究指出，在27例*EGFR*突变阴性NSCLC合并脑转移患者中，18.5%（5/27）的患者存在脑转移症状，主要表现为头痛、呕吐及运动感觉障碍，部分表现为失语、视力下降等。然而，Baek等^[13]^的研究发现在40例*EGFR*突变阴性的脑转移患者中，合并脑转移症状发生率可高达57.5%（23/40），但该研究未对具体症状进行描述。目前缺乏*EGFR*突变阴性NSCLC脑转移患者发病症状的大样本数据，小样本研究显示患者脑转移症状相对较低，多以颅外症状就诊。

## 7 全身治疗疗效

对于*EGFR*突变阴性的脑转移患者，目前可选择的治疗包括放射治疗、手术治疗、化学治疗和免疫治疗。

与放疗比较，传统化疗在脑转移的治疗中效果较差，颅内总缓解率（overall response rate, ORR）非常低。近年来，培美曲塞治疗NSCLC脑转移显示出细微优势，一项小样本的Ⅱ期临床研究^[21]^指出培美曲塞联合顺铂一线治疗无症状脑转移的NSCLC患者的颅内病灶、颅外病灶以及整体的ORR分别为41.9%、34.9%和34.9%，中位无进展生存期（progression-free survival, PFS）和总生存期（overall survival, OS）分别为4.0个月和7.4个月。上述结果提示培美曲塞在NSCLC伴脑转移患者具有一定疗效，但无更多随机对照研究进一步证实。Tian等^[22]^在培美曲塞+顺铂的基础上联合抗血管靶向药物贝伐珠单抗，结果显示联合组与单纯化疗组的颅内ORR分别为53.8% *vs* 44.4%（*P*=0.445），颅内PFS分别为24.3个月vs 10.9个月（*P*=0.008），整体的PFS分别为9.2个月vs 8.2个月（*P*=0.026），中位OS无差异，均为21个月（*P*=0.460），*EGFR*突变阴性患者与阳性患者结果类似。该研究提示贝伐珠单抗可提高脑转移患者的PFS，但未改善整体OS。另一方面，RTOG-0320比较了全脑放疗（whole brain radiation therapy, WBRT）+立体定向放射外科治疗（stereotactic radiosurgery, SRS） *vs* WBRT+SRS+替莫唑胺vs WBRT+SRS+厄洛替尼治疗NSCLC合并1个-3个脑转移的疗效^[23]^，由于入组困难提前结束，可供分析的数据显示三组中位OS分别为13.4个月、6.3个月和6.1个月，中位颅内PFS分别为8.1个月、4.6个月和4.8个月，3级-5级毒副反应发生率分别为11%、41%和49%。从有限的数据来看，在放疗基础上联合血脑屏障通透性好的化疗药物替莫唑胺也并未改善患者的生存，甚至毒副反应发生率高。因此，化学治疗在*EGFR*突变阴性NSCLC伴脑转移治疗中的价值有限。

近些年来，免疫治疗在NSCLC脑转移患者中展现出一定的前景。Crino等^[24]^进行了一项程序性细胞死亡受体1（programmed cell death receptor 1, PD-1）抑制剂Nivolumab二线及后线治疗无症状或脑转移已控制的非鳞NSCLC患者的真实世界研究，入组了409例患者，结果提示ORR和疾病控制率（disease control rate, DCR）分别为17%和39%，PFS和OS分别为3.0个月和8.6个月，3级-4级毒副反应发生率为7%。另外，一项评估了Pembrolizumab（10 mg/kg, q2w）在未经治疗或进展的NSCLC脑转移患者疗效的Ⅱ期临床试验显示：程序性细胞死亡配体1（programmed cell death ligand 1, PD-L1）表达阳性患者的颅内ORR为29.7%[4例完全缓解（complete response, CR），2例部分缓解（partial response, PR）]，整体ORR为18.9%，中位OS为9.9个月，整体和颅内的中位PFS分别为1.9个月和2.3个月，1年和2年OS为40%和34%，但PD-L1阴性者疗效较差，有效率为0%^[25]^。OAK研究^[26]^的亚组分析也比较了PD-L1抑制剂Atezolizumab与多西他赛治疗肺癌脑转移的疗效，结果提示前者有改善OS的趋势（16.0个月vs 11.9个月）。此外，Kotecha等^[27]^回顾性分析了150例接受SRS和PD-1/PD-L1抑制剂治疗的脑转移患者（纳入99例NSCLC），指出免疫抑制剂联合SRS疗效显著，颅内ORR和DCR分别为73%和91%，3个月、6个月和12个月的持续有效率分别为76%、51%和29%，其中SRS同步免疫抑制剂者（±1个半衰期内）效果最佳（CR率：50%，12个月持续有效率：90%）。总体而言，单药免疫治疗在NSCLC脑转移患者中疗效欠佳，尤其是PD-L1阴性患者，与脑部放疗联合可提高颅内控制率，但整体疗效有待进一步提高。

## 8 脑转移疗后颅内病情演变

Yang等^[28]^对42例*EGFR*突变阴性和79例*EGFR*突变阳性NSCLC脑转移接受脑部SRS的颅内病情变化进行了分析，所有患者均接受SRS治疗，其中98%的患者为单次SRS治疗，只有3例患者接受了2次-3次治疗，在78%的中位等剂量线处，中位处方边缘剂量为20 Gy（范围：12 Gy-26 Gy）。结果显示阴性患者的颅内CR率（16% *vs* 38%, *P*=0.01）和ORR（50% *vs* 72%, *P*=0.01）低于阳性患者，*EGFR*突变阴性和阳性患者的12个月和18个月的局部控制率分别为90.8% *vs* 93.9%和85.5% *vs* 92.1%，中位新发脑转移时间分别为10.5个月vs 17个月（*P*=0.02），中位颅内PFS分别为10.5个月vs 17.6个月（*P*=0.02）。Parikh等^[29]^也发现在15例*EGFR*突变阴性/*KRAS*突变阳性、43例*EGFR*突变阴性/*KRAS*突变阴性和9例*EGFR*突变阳性/*KRAS*突变阴性初诊的NSCLC脑转移患者中，颅内病灶SRS治疗后2年的局部控制率分别是66.7%、97.2%和100.0%，1年的颅内远处转移控制率分别是30.0%、73.7%和66.7%（*P*=0.039），颅内中位远处转移时间分别是7.6个月、未达到和未达到。上述研究提示*EGFR*突变阴性脑转移患者接受脑部放疗后局部控制可，但持续时间较短，特别是*EGFR*突变阴性/*KRAS*突变阳性的患者。Barlesi等^[21]^的研究指出，培美曲塞联合卡铂一线治疗42例脑转移性NSCLC的颅内和颅外CR率分别为2.3%和0%，其中41.8%的患者颅内有效，34.9%的患者颅外有效，总ORR为34.9%。另外，13.9%为颅内进展，18.6%为颅外进展，总疾病进展率为25.6%，中位颅内进展时间为5.7个月（95%CI: 4.0-7.6）。Tian等^[22]^的研究进一步表明培美曲塞联合贝伐珠单抗可降低颅内失败率，与不联合方案（11.1%）相比，颅内进展率仅为3.8%，1年和2年的颅内PFS率分别为80.1% *vs* 40.1%和60.1% *vs* 13.2%。上述研究提示，培美曲塞方案颅内外有效率相似，联合贝伐珠单抗可降低颅内失败率，延长颅内控制时间。Goldberg等^[25]^分析了Pembrolizumab治疗脑转移患者的疾病变化情况，在37例PD-L1表达阳性患者的颅内ORR为29.7%（11/37），失败率为43.2%（16/37），18.9%为颅内外均有效，29.7%为颅外有效，1年的颅内PFS为33%。在2例颅内外病灶均可评估的患者中，11%（3/27）的患者颅外有效，但颅内病灶进展，另外11%（3/27）正好相反，表现为颅内有效，但颅外进展。此外，中位颅内起效时间为1.8个月，颅内持续有效时间为5.7个月，颅外持续缓解时间为6.9个月。Kotecha等^[27]^进一步分析了免疫抑制剂联合SRS治疗脑转移灶的疗效，同步免疫抑制剂的中位、3个月、6个月和12个月的颅内最佳肿瘤退缩分别为67%、100%、100%和100%，序贯免疫抑制剂的中位、3个月、6个月和12个月的颅内最佳肿瘤退缩分别为57%、63%、75%和75%，同步组优于序贯组。3个月、6个月和12个月的颅内CR率，同步组亦优于序贯组，分别为82% *vs* 75%（*P*=0.012）、85% *vs* 74%（*P*=0.002）和86% *vs* 72%（*P*=0.005）。两组的颅内进展率相似，分别为9% *vs* 10%。上述研究提示单纯免疫治疗在脑转移患者中颅内失败率高、控制时间短，较少颅内外均有效，联合局部放疗可显著降低颅内失败率，但全身疗效不祥。

## 9 总结

如表 1所示，*EGFR*突变阴性NSCLC脑转移患者预后较差，患者初诊脑转移发生率与*EGFR*突变阳性患者类似，但累积脑转移发生率较阳性患者低。*EGFR*突变阴性患者脑转移发生时间与阳性患者相似。最常见的转移部位是额叶和顶叶，且数目相对局限，多≤3个，较少出现多发病灶和颅内症状，但脑转移灶大小及瘤周水肿范围相对较大。新型的化疗药培美曲塞具有一定颅内疗效，联合抗血管靶向药物贝伐珠单抗可提高脑转移患者的颅内有效率和控制时间，但不能改善总生存。单药免疫抑制剂疗效有限，与脑部放疗联合可以提高颅内有效率和控制率，但整体疗效有待进一步探索。未来在免疫治疗兴起的年代，局部治疗技术以及全身与局部治疗的联合等方面值得进一步探讨。

**表 1:** *EGFR*突变阴性与突变阳性NSCLC的脑转移临床特点 Clinical features of brain metastases between *EGFR* mutation negative and *EGFR* mutation positive NSCLC

Index	Incidence at diagnosis	3-year cumulative incidence	Onset time (median)	Common site	Number (median)	Size (median)	Incidence of CNS symptoms	CNS ORR of drug	CNS PFS (median)
*EGFR* mutation negative	24.6%-25.1%	28.2%-39.2%	7 mon-14 mon	Frontal lobe and parietal lobe	2-3	5.6 mm-11 mm	18.5%-57.5%	15.0%-53.8%	2.3 mon-24.3 mon
*EGFR* mutation positive	26.0%-30.6%	39.2%-46.2%	9.6 mon-19 mon	Frontal lobe and parietal lobe	4-5	4.8 mm-7.1 mm	7.7%-37.8%	43.0%-70.0%	10 mon-NR
EGFR: epidermal growth factor receptor; CNS: central nervous system; ORR: overall response rate; PFS: progression-free survival; NR: not reached; NSCLC: non-small cell lung cancer.									
